# Posterior-Only Approach for Management of Complete Posterior Displaced Type II Odontoid Fracture

**DOI:** 10.1155/2024/8473999

**Published:** 2024-10-24

**Authors:** Seyed Reza Mousavi, Majid Reza Farrokhi, Hamid Jangiaghdam, Mohammadhadi Amir Shahpari Motlagh

**Affiliations:** ^1^Department of Neurosurgery, Shiraz University of Medical Sciences, Shiraz, Iran; ^2^Shiraz Neuroscience Research Center, Shiraz University of Medical Sciences, Shiraz, Iran

## Abstract

**Background:** Odontoid fracture (OF) is one of the most common spinal fractures. Type II in D'Alonzo's classification is still the most common and should be considered unstable unless proven otherwise. Thus, surgical stabilization has received significant attention. Although posterior displacement is common in type II OF, complete displacement is extremely rare, and very few reports are available in the literature.

**Case Presentation:** We report the case of a 60-year-old man with acute type II OF with complete posterior displacement and myelopathy. The patient was managed utilizing a posterior-only single approach for reduction and stabilization.

**Conclusion:** Posterior displacement of type II OF has been traditionally managed with close reduction and anterior or posterior stabilization. Closed reduction in cases of complete posterior displacement carries a significant risk of neurologic deterioration. Anterior, combined, and posterior approaches have been taken for this condition. The posterior-only approach in experienced hands has the least mortality and morbidity with at least the same neurologic and fusion outcomes.

## 1. Introduction

Odontoid fracture (OF) is the most common cervical spinal fracture in elderly people and also the most common form of C2 fracture [1]. It has three main types, according to D'Alonzo's classification, with type II being the most common [2]. Type II OF is also the most challenging form for management. Before planning a treatment method for such fractures, several considerations must be taken, including the patient's age, comorbidities, bone quality, fracture shape, and displacement [1, 2]. Among these, physical features of the fracture have been the subject of many studies; some correlate the angle and displacement as major factors affecting our decision [3]. Posterior displacement occurs mostly when the fracture line is anterosuperior to the posteroinferior of the dense process [3, 4]. Yet, there are a couple of complete posterior displacement case reports [4, 5]. Herein, we present our case of complete posterior displacement of type II OF with a single posterior cervical approach.

## 2. Case Presentation

**Figure 1:**
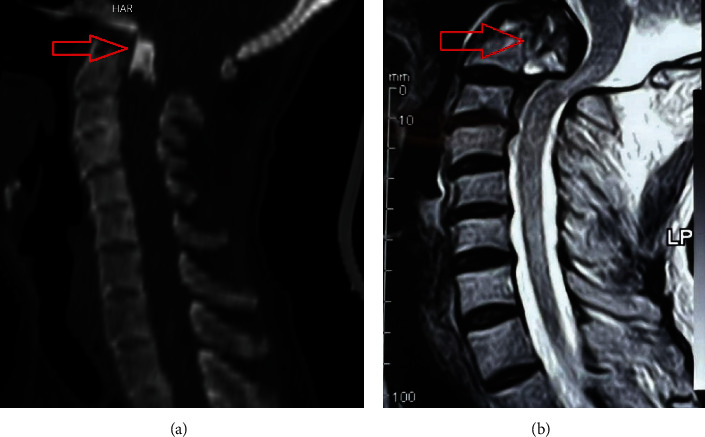
Preoperational midsagittal cervical spinal CT (a) and T2 weighted sequence-MRI (b) showing complete posterior displacement of odontoid process (arrows). CT, computed tomography; MRI, magnetic resonance imaging.

A 60-year-old man, following a motor vehicle accident, presented to our center with neck pain and weakness. A complete neurologic examination revealed that all muscle powers were 3/5. After cervical spine computed tomography (CT) and magnetic resonance imaging (MRI) (Figure 1), we diagnosed type II OF with complete posterior displacement. Spinal cord compression and grade C of the American Spinal Injury Association (ASIA) myelopathy grading system were documented.

**Figure 2:**
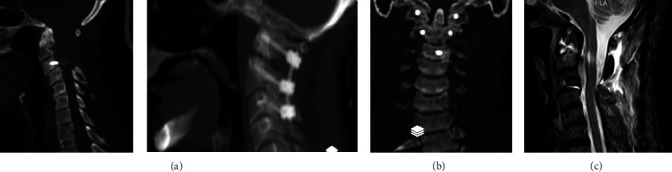
Postoperational midsagittal (a), coronal (b), parasagittal (c) cervical spine CT-scan and midsagittal T2 weighted cervical spine MRI, showing complete reduction and fusion of odontoid fracture. CT, computed tomography; MRI, magnetic resonance imaging.

After the patient and his close relatives provided consent, surgical management was provided for his treatment. After general anesthesia, intraoperative neuromonitoring was provided for him from before prone positioning until the end of the procedure and repositioning. In the standard prone position, a midline skin incision was made from the external occipital protuberance to the C3 spinous process. After muscle and soft tissue dissection, bony landmarks were exposed from the suboccipital area to the C3 lamina. At first, C1 and C2 bilateral laminectomy was performed. Then, C1 lateral mass and C2 pedicle screws were inserted under the guide of fluoroscopy. Cervical traction was then applied gradually using a Garner head clamp. This was along with unilateral temporary rod insertion and gradual atlantoaxial distraction. This process was done under the guidance of fluoroscopy and neurophysiologic monitoring till near-complete reduction was achieved. Then, lateral mass screws were inserted in the C3 lateral masses, and C1–C3 fixation was done (Figure 2).

Minerva brace was applied for the patient for the next 4 months. So far, nearly 8 months in post-op period, the patient has had nearly a 100 sessions of physical therapy and significant neurologic improvement is obvious.

**Figure 3:**
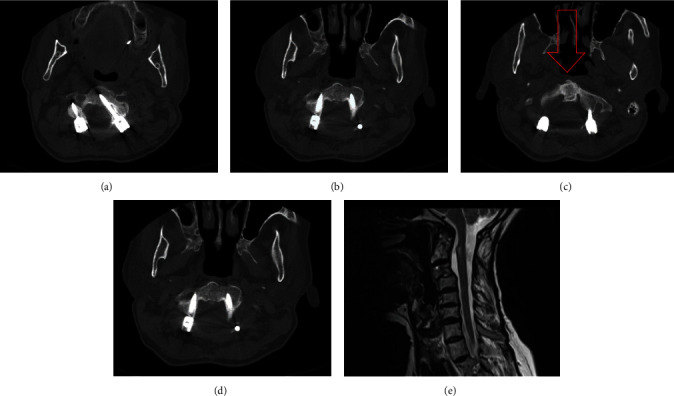
Cervical spinal CT-scan (a–d) and MRI (e) in 1-year follow-up, showing good fusion of odontoid process with stable position (arrow). CT, computed tomography; MRI, magnetic resonance imaging.

In his 1-year follow-up, no significant disability was observed and daily activities were not grossly limited due to neurologic deficit. Radiologic investigations were performed with cervical spine CT-scan and MRI (Figure 3). Besides good fusion and stable spinal segment, no canal compromise or compression on neural tissue was observed.

## 3. Discussion

OF has three types based on D'Alonso's classification; type II is the most common and the most challenging to manage [1, 2]. Displacement, angulation, and comminution make it more difficult to treat [3, 4]. This fracture has a bimodal age distribution, and treatment modalities differ between old and young patient groups [1–3]. In elderly patients, due to the higher chance of nonunion, early surgical intervention is acceptable [6]. In young patients (<60 years old), if there is no atlantoaxial Joint dislocation (AAD), closed reduction and external immobilization are widely utilized. Exceptions are those with nonunion or failure of nonsurgical treatment, angulation (>10°), displacement (>5 mm), and comminution of the odontoid process (OP) [1, 7, 8].

Displacement has been the subject of many studies. When displacement is minor, closed reduction followed by external immobilization is standard [1–4, 6, 7, 9]. The anterior odontoid screw can also be the initial treatment plan for displaced OF after acceptable reduction [7]. For nonreducible OFs, however, open reduction is mandatory before surgical stabilization [1, 2]. Anterior displacement can be reduced through the anterior (transoral/trans facial) or posterior approach (PA) [4, 5]. Reduction of posterior displacement (>5 mm) is mainly via closed traction [1, 4]. Yet, the reduction of complete posterior displacement—which is extremely rare—is discussed well in Rathod et al.'s [4] study. Due to severe neurological deficits (respiratory arrest), closed reduction in such cases carries a great risk. In Wang, Zhang, and Xu's [10] study, open reduction through a transoral approach was utilized. As we know, open reduction is followed by fusion; another anterior or PA should be taken if transoral reduction is utilized. In such conditions, complications and morbidities would accumulate. Therefore, open reduction and fusion via one approach are preferred.

Fusion procedures for OFs consist of an anterior approach (AA) for the odontoid screw and a PA for atlantoaxial fusion. These approaches have been reviewed in many aspects. In a recent review by Texakalidis et al. [11], contraindications for AA were mentioned as comminuted fractures, cervicothoracic kyphosis, severe osteoporosis, transverse ligament rupture, late fractures, and a fracture line that is not anterosuperior to posterosuperior. Severe obesity is also considered a relative contraindication. In this study, the fusion rate was greater in PA, while AA had higher rates of reoperation and technical error [11].

Considering our experience with the posterior-only approach in the craniovertebral junction [1, 12–14], along with Rathod's et al. [4] study, we utilized the posterior-only approach to manage our case. In the postoperative course, myelopathy improved significantly. After 2 months of follow-up, no significant complications or morbidities were noted.

## 4. Conclusion

Type II OF is complicated to manage. The fracture line, angle, and displacement are some of the most significant features that affect management planning. Significant posterior displacement has been traditionally managed with closed reduction and anterior (odontoid screw) or posterior stabilization. Yet, closed reduction in cases of complete posterior displacement carries a significant risk of neural tissue damage due to severe spinal canal narrowing. Anterior, combined, and PAs have been taken for this condition. In experienced hands, the posterior-only approach has the least mortality and morbidity with at least the same outcome as other approaches. Besides favorable fusion, neurologic improvement is noticed in such an approach if available.

## Data Availability

The data of this publication are available offline and those who interest, may contact the authors and get access to all these data.
